# Toripalimab, a therapeutic monoclonal anti-PD-1 antibody with high binding affinity to PD-1 and enhanced potency to activate human T cells

**DOI:** 10.1007/s00262-024-03635-3

**Published:** 2024-02-24

**Authors:** Narendiran Rajasekaran, Xiaoguang Wang, Sruthi Ravindranathan, Daniel J Chin, Su-Yi Tseng, Scott L Klakamp, Kate Widmann, Varun N Kapoor, Vladimir Vexler, Patricia Keegan, Sheng Yao, Theresa LaVallee, Sanjay D Khare

**Affiliations:** 1 Coherus Biosciences, 333 Twin Dolphin Drive, Suite 600, Redwood City, CA 94065 USA; 2 TopAlliance Biosciences, 9430 Key West Ave, Suite 125, Rockville, MD 20850 USA; 3 Shanghai Junshi Biosciences, Shanghai, China

**Keywords:** Toripalimab, Immune checkpoint inhibitor, PD-L1 status irrespective, FG loop, PD-1 signaling

## Abstract

Over the past decade, US Food and Drug Administration (FDA)-approved immune checkpoint inhibitors that target programmed death-1 (PD-1) have demonstrated significant clinical benefit particularly in patients with PD-L1 expressing tumors. Toripalimab is a humanized anti-PD-1 antibody, approved by FDA for first-line treatment of nasopharyngeal carcinoma in combination with chemotherapy. In a post hoc analysis of phase 3 studies, toripalimab in combination with chemotherapy improved overall survival irrespective of PD-L1 status in nasopharyngeal carcinoma (JUPITER-02), advanced non-small cell lung cancer (CHOICE-01) and advanced esophageal squamous cell carcinoma (JUPITER-06). On further characterization, we determined that toripalimab is molecularly and functionally differentiated from pembrolizumab, an anti-PD-1 mAb approved previously for treating a wide spectrum of tumors. Toripalimab, which binds the FG loop of PD-1, has 12-fold higher binding affinity to PD-1 than pembrolizumab and promotes significantly more Th1- and myeloid-derived inflammatory cytokine responses in healthy human PBMCs in vitro. In an ex vivo system employing dissociated tumor cells from treatment naïve non-small cell lung cancer patients, toripalimab induced several unique genes in IFN-γ and immune cell pathways, showed different kinetics of activation and significantly enhanced IFN-γ signature. Additionally, binding of toripalimab to PD-1 induced lower levels of SHP1 and SHP2 recruitment, the negative regulators of T cell activation, in Jurkat T cells ectopically expressing PD-1. Taken together, these data demonstrate that toripalimab is a potent anti-PD-1 antibody with high affinity PD-1 binding, strong functional attributes and demonstrated clinical activity that encourage its continued clinical investigation in several types of cancer.

**Supplementary Information:**

The online version contains supplementary material available at 10.1007/s00262-024-03635-3.

## Introduction

Immune checkpoint inhibitors (ICIs) targeting programmed cell death 1 (PD-1) and programmed cell death ligand 1 (PD-L1) have revolutionized cancer treatment in the recent years, affording long-term survival benefit in a broad range of cancer patients. PD-1 is an inhibitory cell surface receptor that is upregulated upon T cell activation. Upon binding to its ligands PD-L1 and PD-L2, that are expressed on antigen presenting cells and/or tumor cells [1], PD-1 recruits the phosphatases SHP1 and SHP2 which in turn suppresses T cell activation and function [2]. While this mechanism of regulating T cell immune response is necessary in maintaining immune tolerance to autoantigens, several tumors over-express PD-L1 in response to inflammatory mediators and downregulate anti-tumor function of T cells leading to tumor immune evasion [3–5]. However, anti-PD-1 monoclonal antibodies by blocking the interaction of PD-1 with its ligands, PD-L1/PD-L2, reinvigorate the anti-tumor T cell responses and enhancing anti-tumor immunity [6].

In the recent years, the US Food and Drug Administration (FDA) has approved several anti-PD-1 monoclonal antibodies (Abs) that include pembrolizumab, nivolumab and cemipilimab for use in more than 50 tumor indications [7]. These PD-1 Abs have a beneficial effect on the treatment of patients with a broad range of advanced or metastatic cancer compared to conventional chemotherapy [8]. In most clinical studies, the PD-L1 status of the tumor correlated with the overall clinical benefit of the PD-1 therapy, whereby PD-L1 positive patients responded better to PD-1 immunotherapy treatment, either alone or in combination with chemotherapy. For instance, pembrolizumab in combination with chemotherapy in PD-L1 high non-small cell lung cancer (NSCLC) patients (TPS > 1%) demonstrated better clinical activity compared to PD-L1 low NSCLC (TPS < 1%) (Keynote 189; PD-L1 screen: 22C3 pharmDx assay) though both PD-L1 groups benefitted from the combination treatment [9]. Similarly, the therapeutic efficacy of nivolumab correlated with the expression levels of PD-L1 in cancer patients (CheckMate: 037; PD-L1 screen: 28-8 pharmDx assay) [10–12]. Furthermore, based on the objective response rate, PD-L1 high NSCLC patients gain more benefits than PD-L1 low patients in response to treatment with cemiplimab in combination with chemotherapy (EMPOWER-Lung 3; PD-L1 screen: 22C3 pharmDx assay) [13]. A similar benefit in the overall population upon treatment with atezolizumab was driven by patients with high PD-L1 expression (IMpower110 PD-L1 screen: 22C3 pharmDx assay) [14]. Thus, there is an unmet need for PD-1 treatment approaches that clinically benefits cancer patients irrespective of their PD-L1 status.

Toripalimab is a PD-1 targeting humanized IgG4 monoclonal Ab that has been recently approved in combination with cisplatin and gemcitabine by US Food and Drug Administration (FDA) for the first-line treatment of adults with metastatic or recurrent locally advanced nasopharyngeal carcinoma (NPC), and as monotherapy for the treatment of adults with recurrent, unresectable or metastatic NPC with disease progression on or after platinum-containing chemotherapy.

In three different phase 3 clinical trials, namely JUPITER-02 (NPC), JUPITER-06 (ESCC) and CHOICE-01 (NSCLC), that toripalimab in combination with chemotherapy was generally efficacious in PD-L1 positive and PD-L1 negative/low cancer patients in subgroup analyses (PD-L1 screen: JS311 IHC assay). The clinical activity of toripalimab/chemotherapy, irrespective of PD-L1 status from this post hoc analysis, prompted us to further investigate the characteristics of toripalimab molecularly and functionally. Considering that prospective clinical studies with appropriate power are required to evaluate the efficacy of toripalimab in the PD-L1 subgroup, we evaluated with these preclinical studies if there are pharmacological parameters of toripalimab that could further support such studies. Pembrolizumab which is approved in the largest number of tumor indications in the PD-1 Ab class and has provided clinical benefit in various solid tumors was used a comparator [15].

## Results

### Toripalimab in combination with chemotherapy improved overall survival in a post hoc analysis of randomized controlled trials

Three randomized controlled phase 3 studies with toripalimab plus chemotherapy were evaluated for overall survival (OS) stratified by PD-L1 levels. Two PD-L1 scoring criteria, tumor proportion score (TPS) and the combined positive score (CPS), were used for post hoc analysis.

**Fig. 1:**
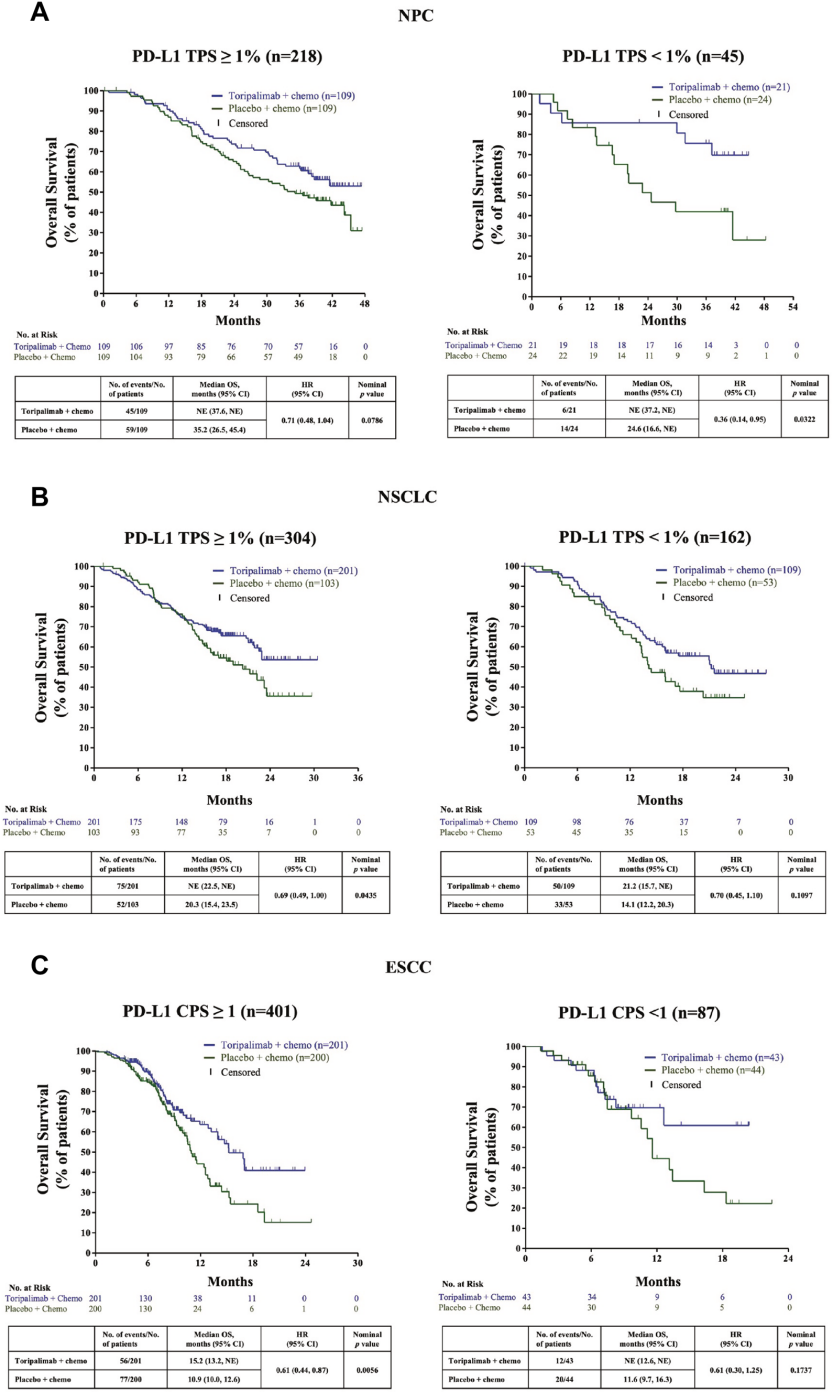
Toripalimab in combination with chemotherapy improved overall survival in a post hoc analysis of randomized controlled trials. Kaplan–Meier estimates of OS are shown to compare the toripalimab plus chemotherapy arm with the placebo plus chemotherapy arm in **A** NPC for PD-L1 TPS ≥ 1% and the PD-L1 TPS < 1% subgroups, **B** NSCLC for PD-L1 TPS ≥ 1% and the PD-L1 TPS < 1% subgroups, (C) ESCC for PD-L1 CPS ≥ 1 and the PD-L1 CPS < 1 subgroups. Censored patients are marked with “┃” in the graph. Numbers of patients at risk at indicated time points are shown below the x axis. Number of events, median OS, hazard ratio for death and nominal *p* values are shown in the table below the Kaplan–Meier curves. CPS, combined positive score; ESCC, esophageal squamous cell carcinoma; HR, hazard ratio; NE, not estimated; NSCLC, non-small cell lung cancer: NPC, nasopharyngeal carcinoma; OS, overall survival; PD-L1, programmed cell death ligand 1; TPS, tumor proportion score

The JUPITER-02 study (Fig. 1A) evaluated patients with nasopharyngeal carcinoma (NPC) randomized to toripalimab plus chemotherapy compared to placebo plus chemotherapy and retrospectively evaluated OS by PD-L1 status as defined by TPS ≥ 1% (*n* = 218) or TPS < 1% (*n* = 45). In the TPS ≥ 1% group, the median OS of the placebo arm was 35.2 months, whereas that of the toripalimab arm had not been reached at the time of data cutoff (18th November 2022). In the TPS < 1% group, the median OS of the placebo arm was 24.6 months, whereas that of the toripalimab arm had not been reached at the time of data cutoff. The hazard ratios (HR) for the PD-L1 high and low subgroups are 0.71 (*P* = 0.0786) and 0.36 (*P* = 0.0322) respectively, demonstrating better OS with toripalimab plus chemotherapy treatment in both PD-L1 subgroups.

The CHOICE-01 study [16, 17] (Fig. 1B) evaluated patients with NSCLC randomized to toripalimab plus chemotherapy compared to placebo plus chemotherapy and retrospectively evaluated OS by PD-L1 status as defined by TPS ≥ 1% (*n* = 304) or TPS < 1% (*n* = 162). In the TPS ≥ 1% group, median OS had not been reached for toripalimab arm at the time of data cutoff (31st October 2021) while the median OS of the placebo arm was 20.3 months, with a HR of 0.69 (*P* = 0.0435). On the other hand, at the time of data cutoff, in the TPS < 1% group, the median OS of the toripalimab arm was 21.2 months, whereas that of the placebo arm was 14.1 months with HR of 0.70 (*P* = 0.1097). In this trial, upon treatment with toripalimab plus chemotherapy, although the OS is significant only in the TPS ≥ 1% subgroup, there is a noteworthy increase in OS in the TPS < 1% subgroup.

The JUPITER-06 study[18] (Fig. 1C) evaluated patients with ESCC randomized to toripalimab plus chemotherapy compared to placebo plus chemotherapy and retrospectively evaluated OS by PD-L1 status as defined by CPS ≥ 1 (*n* = 401) or CPS < 1 (*n* = 87). In the CPS ≥ 1% group, at the time of data cutoff (March 22, 2021), the median OS of 15.4 months in the toripalimab arm is significantly better than placebo arm of 10.9 months (HR 0.61; *P* = 0.0056). On the other hand, in the CPS < 1 group, the OS of the toripalimab arm was not significantly better than the placebo (HR 0.61; *P* = 0.1737). However, like the CHOICE-01 study, the improvement in OS with toripalimab plus chemotherapy in comparison with placebo plus chemotherapy in both CPS ≥ 1 and CPS < 1 subgroups needs to be noted. Taken together, the above phase 3 clinical trials provide evidence that toripalimab in combination with chemotherapy conferred better OS than chemotherapy alone in first-line treatment of patients with advanced and/or metastatic NPC, NSCLC or ESCC irrespective of PD-L1 expression.

### Toripalimab exhibits a high binding affinity for PD-1

**Fig. 2:**
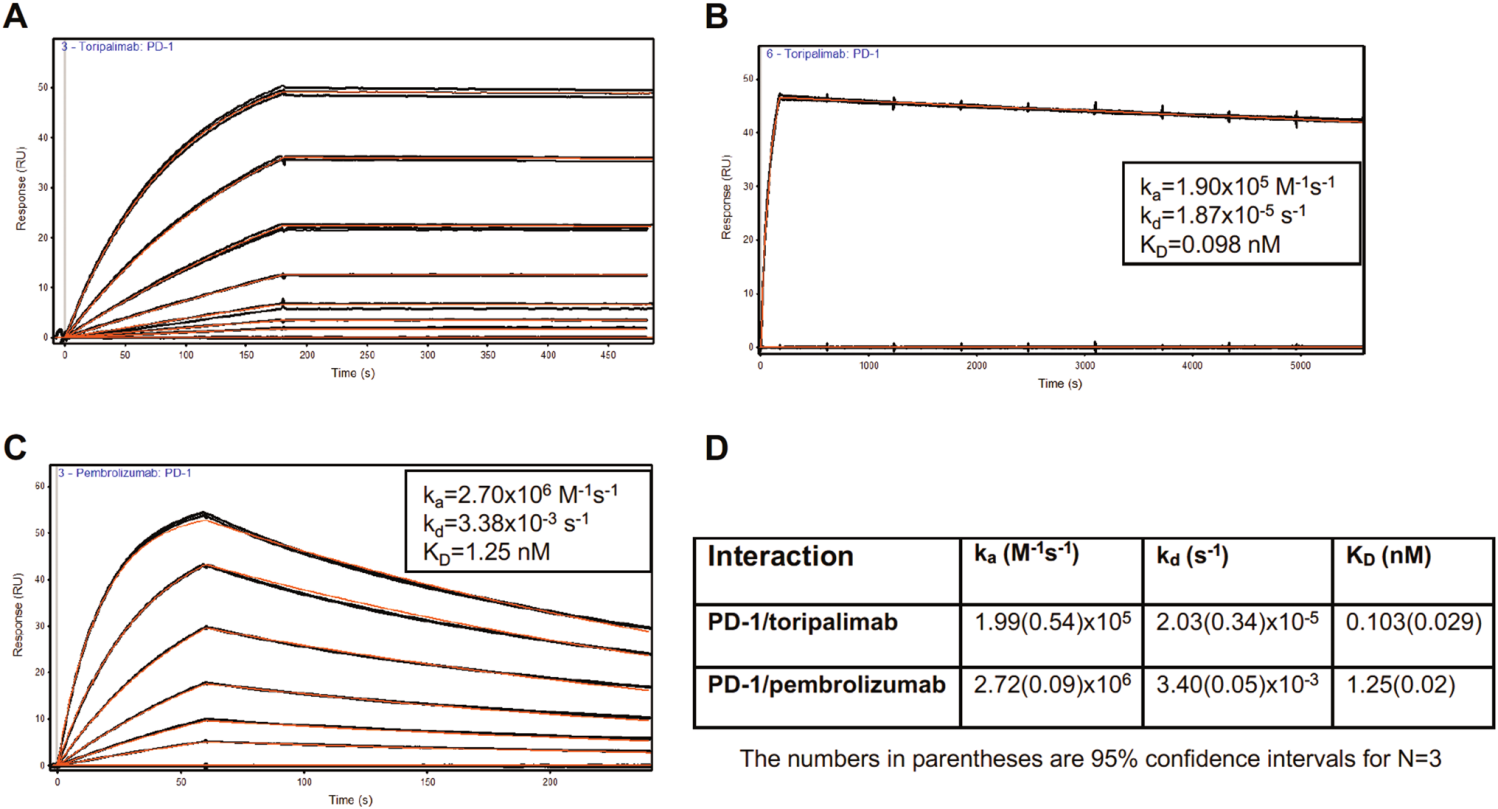
Representative SPR analysis of human PD-1 binding to PD-1 antibodies. **A** Biacore sensorgrams of PD-1 binding to covalently immobilized toripalimab. PD-1 was injected in triplicate for 3 min in a range from 0.93 to 59.5 nM with dissociation followed for 5 min. **B** PD-1 at the highest concentration of 59.5 nM was injected in triplicate for 3 min with dissociation followed for 90 min. **C** Sensorgrams of PD-1 binding to covalently immobilized pembrolizumab. PD-1 was injected in triplicate for 1 min in a range from 0.63 to 20.3 nM with dissociation followed for 3 min. The equilibrium dissociation constants and kinetic rate constants for each interaction are noted in the panels. All sensorgrams were globally fit (red lines) to a 1:1 interaction model including a term for mass transport. **D** Average kinetic rate and equilibrium dissociation constants (*k*_*a*_, *k*_*d*_ and *K*_*D*)_ from three replicate experiments for PD-1 binding to toripalimab and pembrolizumab

To evaluate the binding kinetics of toripalimab to human PD-1, surface plasmon resonance (SPR) assay was performed with the toripalimab immobilized on C1 sensor chips. The human PD-1 protein was flowed across the PD-1 Ab at various concentrations. Biacore sensograms of PD-1 binding to covalently immobilized toripalimab (Fig. 2A and Supplementary Fig. S1A) were compared to pembrolizumab (Fig. 2C and Supplementary Fig. S1B). Since we observed a very slow dissociation of PD-1 from toripalimab, more extensive dissociation data were generated for toripalimab with additional injections at the highest antigen concentration followed by a longer dissociation phase (Fig. 2B). Toripalimab showed 12-fold higher affinity for PD-1 compared to pembrolizumab, 0.103 versus 1.25 nM, respectively. The association rate constant *k*_a_, dissociation rate constant *k*_d_ and equilibrium dissociation constant *K*_D_ for pembrolizumab and toripalimab are listed in Fig. 2D. The higher binding affinity of toripalimab is explained by its dissociation rate constant (*k*_d_) that is 168-fold slower than pembrolizumab, 2.03 × 10^−5^ versus 3.40 × 10^–3^ s^−1^, respectively (Fig. 2D). Together, these data demonstrate that toripalimab shows significant differences compared to pembrolizumab in its binding kinetics and affinity to human PD-1.

### Toripalimab elicits strong T cell activation in SEB-activated PBMCs

**Fig. 3:**
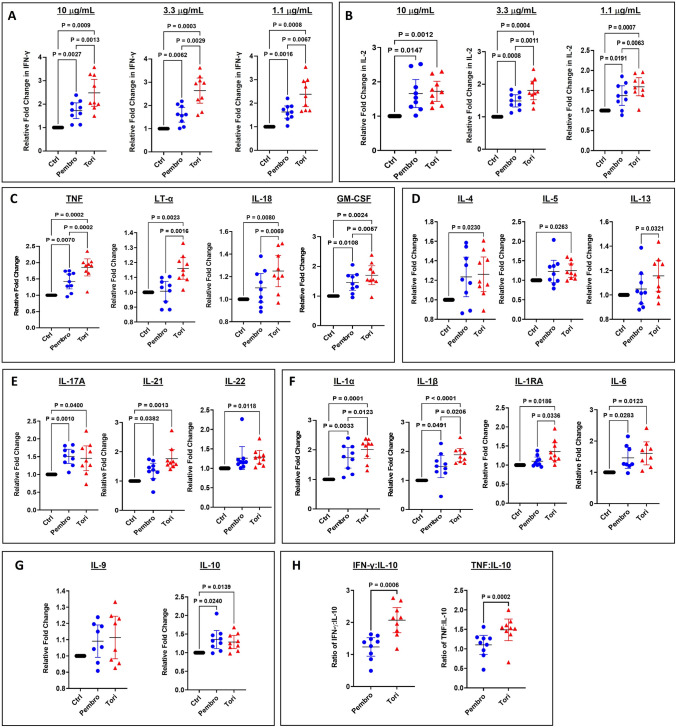
Toripalimab is more potent than pembrolizumab in enhancing levels of Th1 cytokines in response to SEB-mediated T cell activation in human PBMCs. PBMCs from nine healthy donors were cultured with 100 ng/mL SEB in the presence of 10, 3.3 or 1.1 µg/mL anti-PD-1 antibody (Ab): pembrolizumab (pembro) or toripalimab (tori) or isotype Ab control (Ctrl) in triplicate. After 3 days, cell supernatants were collected to examine IFN-γ (**A**) and IL-2 (**B**) levels by ELISA. Graphs indicate relative fold change (mean ± SEM, *n* = 9) in cytokine secretion in the presence of pembro or tori relative to Ctrl. Supernatants from PBMCs cultured with 100 ng/mL SEB in the presence of 3.3 µg/mL Ab: pembro or tori or Ctrl in triplicate were tested by performing Luminex assays to examine Th1 cytokines (**C**), Th2 cytokines (**D**), Th17 cytokines (**E**), myeloid-derived cytokines (**F**), IL-9, IL-10 (**G**) and ratios of IFN-γ/IL-10 and TNF-α/1L-10 (**H**). Each point in the graphs represents an individual donor, Mean ± SEM, *n* = 9. Statistical analysis was performed using one-way ANOVA with Tukey’s multiple comparisons tests for A-G and using paired t test for H. *p* < 0.05 is considered significant. *p* values of significance are shown

Assessing the mechanism of action, the ability of toripalimab and pembrolizumab, to promote T cell activation was evaluated. Human peripheral blood mononuclear cells (PBMCs) from 9 independent healthy human adult donors were stimulated with Staphylococcal enterotoxin B (SEB) [19], and the response to T cell stimulation upon treatment with PD-1 Abs toripalimab and pembrolizumab was quantified by measuring the release of the Th1 cytokines IFN-γ and IL-2 by ELISA. To minimize the variations across donors, the effects of the two PD-1 Abs were expressed as fold changes of cytokine production. All fold changes referred to in this section were mean fold changes in cytokine expression calculated over control Ab treatment. As expected, the addition of either toripalimab or pembrolizumab at three different concentrations (10, 3.3 and 1.1 μg/mL) led to statistically significant higher levels of IFN-γ secretion compared to control (Fig. 3A). Interestingly at 10 μg/mL of PD-1 Ab treatment, toripalimab induced a significantly larger increase in IFN-γ secretion compared to pembrolizumab (2.5-fold and 1.5-fold, respectively, *P* = 0.0013) (Fig. 3A). Similarly, at the lower concentrations of Ab treatments 3.3 and 1.1 μg/mL, toripalimab was able induce significantly higher levels of IFN-γ than pembrolizumab (*P* = 0.0029 and 0.0067, respectively) (Fig. 3A). Upon testing for IL-2 expression, though both toripalimab and pembrolizumab were able to induce significantly higher IL-2 secretion at all three Ab concentrations, toripalimab was significantly more potent than pembrolizumab at 3.3 μg/mL (*P* = 0.0011) and 1 μg/mL (*P* = 0.0063), and demonstrated maximum IL-2 secretion at 3.3 μg/mL (Fig. 3B).

To examine the effect of the PD-1 Ab treatments on a broad range of cytokines, the supernatants were further analyzed by performing multiplex Luminex assays. As demonstrated in Fig. 3B, toripalimab treatment resulted in maximum IL-2 secretion that was more potent than pembrolizumab treatment at 3.3 μg/mL, while pembrolizumab induced maximum IL-2 secretion at 10 μg/mL with a fold change similar to toripalimab treatment (Supplementary Table ST1). Thus, for this assay supernatants collected from 3.3 μg/mL treatment groups were chosen to best differentiate toripalimab from pembrolizumab. We grouped the cytokines tested into five categories based on their function: Th1, Th2, Th17, Th9, suppressive and myeloid-associated cytokines. Among the Th1 cytokines (Fig. 3C), tumor necrosis factor (TNF), a critical cytokine that is required for an anti-tumor immune response, was induced at significantly higher levels by toripalimab (1.85-fold) than pembrolizumab (1.42-fold). Similarly, secretion of granulocyte macrophage colony-stimulating factor (GM-CSF) was significantly enhanced by toripalimab (1.70-fold) compared to pembrolizumab (1.46-fold). While treatment with pembrolizumab did not increase the expression of the Th1 cytokines IL-18 (1.08-fold) and lymphotoxin alpha (LT-*α*) (1.0-fold), toripalimab induced a modest but significant increase in secretion of IL-18 (1.25-fold) and a small increase in LT-*α* (1.15-fold) (Fig. 3C). Of all the Th1 cytokines we analyzed, toripalimab induced the highest magnitude of change in the secretion of IFN-γ followed by TNF and IL-2. Taken together, our data demonstrate toripalimab treatment resulted in a greater overall Th1 response that was stronger in magnitude compared to pembrolizumab. In case of Th2 cytokines IL-4, IL-5 and IL-13 tested [20] (Fig. 3D), both toripalimab and pembrolizumab induced comparable levels of IL-4 (1.26-fold and 1.23-fold, respectively) and IL-5 (1.24-fold and 1.22-fold, respectively). However, only toripalimab was able to induce a small but significant increase of IL-13 (1.15-fold) in comparison with pembrolizumab (1.04-fold).

Upon characterizing the impact of toripalimab and pembrolizumab on secretion of Th17-associated cytokines (Fig. 3E) [21], both toripalimab and pembrolizumab induced comparable increases in levels of expression of IL-17A (1.5-fold and 1.45-fold, respectively) and negligible changes in IL-22. Secretion of IL-21 was increased more by toripalimab (1.76-fold) compared to pembrolizumab (1.38-fold) though the differences were statistically insignificant.

Proinflammatory cytokines such as IL-6, IL-1α, IL-1β and IL1RA are primarily secreted by activated myeloid cells and play critical roles in an anti-tumor immune response [22, 23] (Fig. 3F).

IL-1α and IL-1β were secreted at significantly higher levels by toripalimab (2.00-fold and 1.88-fold, respectively) compared to pembrolizumab (1.73-fold and 1.48-fold, respectively). Interestingly, IL-1RA, the antagonist [24] that prevents the binding of IL-1 protein to its receptor and inhibits its downstream signaling, was induced only by toripalimab (1.35-fold). Comparable increases in the expression of IL-6 by toripalimab (1.60-fold) and pembrolizumab (1.46-fold) were observed. In summary, toripalimab was more potent than pembrolizumab in inducing myeloid-derived cytokines IL-1α, IL-1β and IL1RA possibly through bystander stimulation of myeloid cells in the PBMCs.

In the evaluation of the Th9-derived cytokine IL-9 [25], no significant increase in its expression was observed in response to PBMC treatment with toripalimab (1.11-fold) or pembrolizumab (1.09-fold) (Fig. 3G). The suppressive cytokine IL-10 was induced at similar levels by toripalimab (1.28-fold) and pembrolizumab (1.35-fold) (Fig. 3G). Despite IL-10 secretion being modest, it may still modulate the immune response depending on the ratio of the inflammatory cytokines to IL-10. To examine whether either of the PD-1 Abs could skew the immune stimulatory to immune suppressive cytokine levels, we analyzed the ratios of IFN-γ to IL-10 and TNF to IL-10 across all the donor PBMCs upon treatment with either toripalimab or pembrolizumab. We observed that in contrast to pembrolizumab, toripalimab skewed the response in favor of inflammatory cytokines (IFN-γ and TNF) (Fig. 3H). The absolute concentrations of cytokines for all donors tested are presented in supplementary Fig. S2.

Taken together, these data demonstrate that toripalimab is more potent than pembrolizumab in activation of T cells in vitro and that it significantly increases the secretion of Th1- and myeloid-derived cytokines, favoring a proinflammatory function.

### Toripalimab enhances CD3/CD28-mediated T cell activation of human naïve CD8 + T cells

**Fig. 4:**
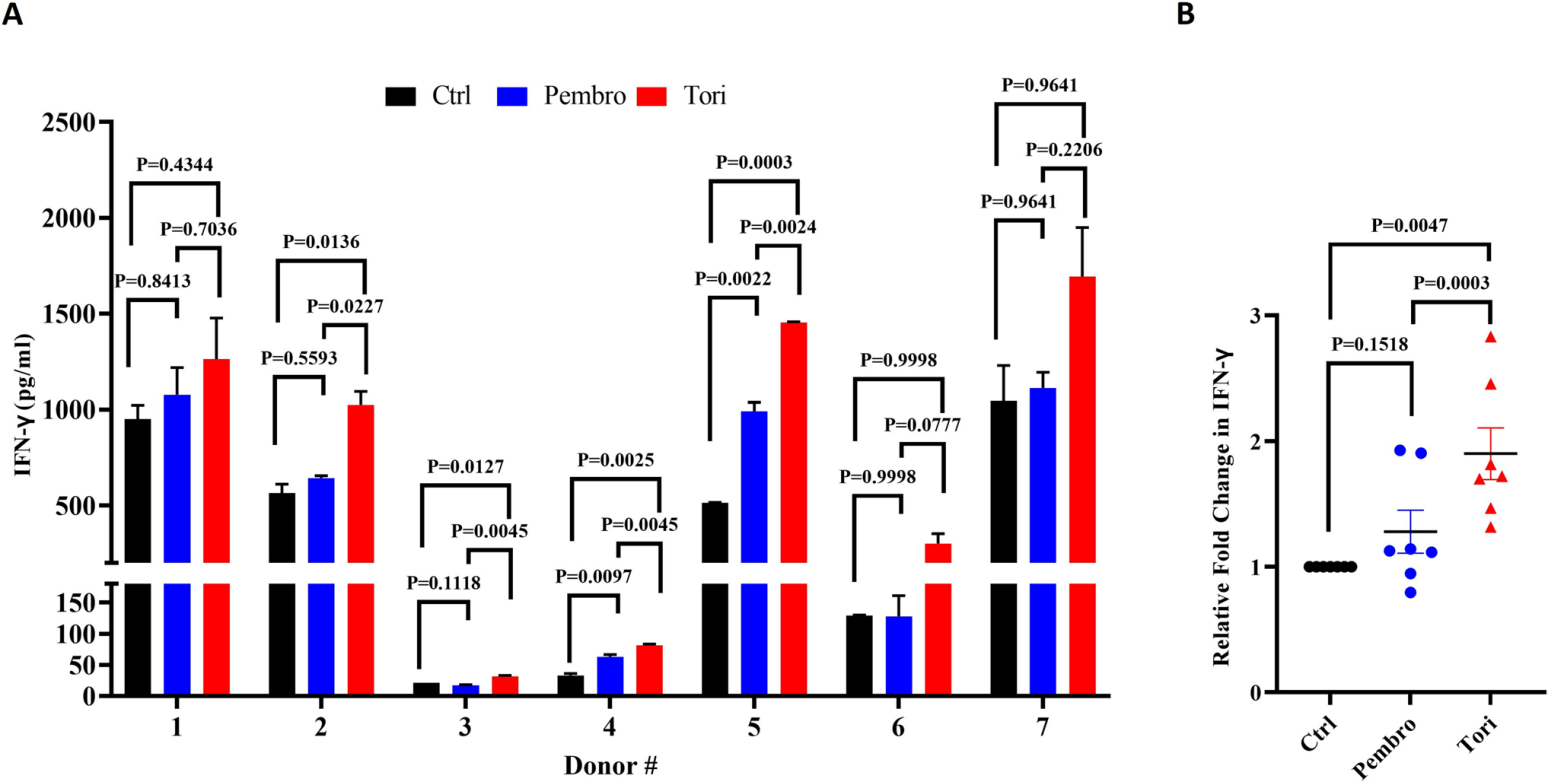
Toripalimab enhances IFN-γ secretion in CD3/CD28-activated naïve human CD8 + T cells. Naïve CD8 + T cells from seven healthy donors were activated with human anti-CD3 (0.5 µg/mL) and human anti-CD28 (0.5 µg/mL) immobilized on the plate surface. 10 µg/mL of isotype control Ab (Ctrl), pembrolizumab (pembro) or toripalimab (tori) in duplicate wells. IFN-γ levels in cell culture supernatant was quantified on day 3 of activation using ELISA. **A** IFN-γ levels from seven donors. **B** Fold change in concentration of IFN-γ relative to the Ctrl. *p* values in A were calculated using one-way ANOVA followed by Tukey’s multiple comparison test and those in B were calculated via paired t test. *P* < 0.05 is considered significant

To investigate the effect of PD-1 Abs in the absence of PD-L1 expressing APCs or tumor cells, human CD8 T cells were isolated from healthy donors and activated with immobilized human anti-CD3 and anti-CD28 Abs in the presence of the PD-1 Abs. Three days after activation, IFN-γ levels were measured in the supernatant, as an indicator of T cell activation. In comparison with control, while pembrolizumab increased levels of IFN-γ in only 2 of the 7 donors, toripalimab did so in all 7 donors, with the differences being significant in four (Fig. 4A). Upon calculating the average fold change in IFN-γ with respect to control, CD8 T cells cultured in the presence of toripalimab showed significantly higher IFN-γ secretion, 1.90-fold, when compared to pembrolizumab, 1.20-fold (Fig. 4B). These data suggest that toripalimab has increased potency to activate CD8 T cells even in the absence of PD-L1 expressing APCs or tumor cells.

### Binding of toripalimab to PD-1 induces lower levels of PD-1 signaling compared to pembrolizumab

**Fig. 5:**
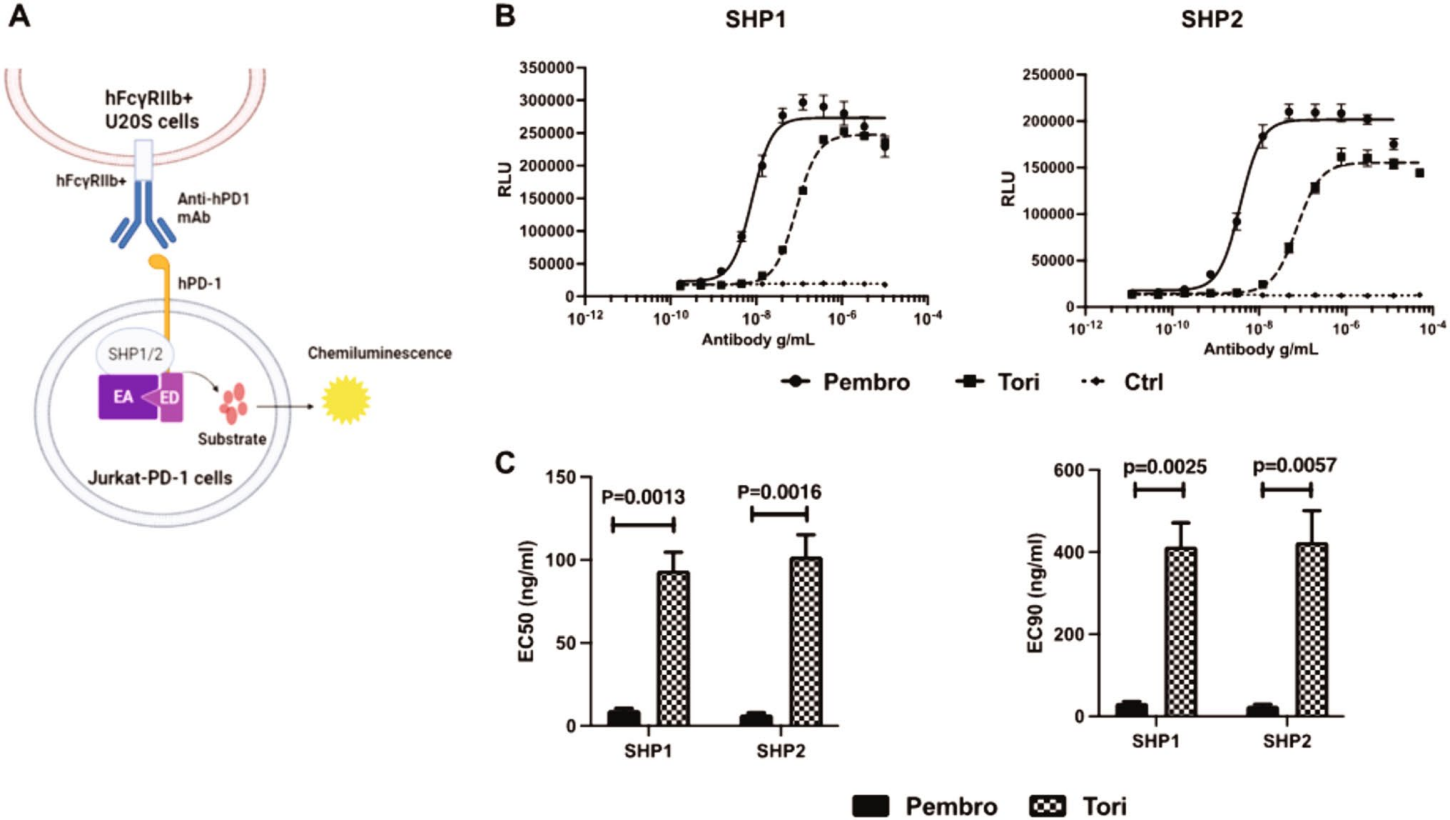
Toripalimab recruits lower levels of SHP1 or SHP2 than pembrolizumab in PathHunter® Jurkat PD-1 cell lines. **A** Schematic representation of the experimental system. PathHunter® Jurkat PD-1 cell lines expressing the SHP1 or SHP2 signaling assay system were cocultured with U2OS cells opsonized with increasing doses of isotype Ab (Ctrl), pembrolizumab (pembro) or toripalimab (tori) (dose range 0.01–10 µg/mL) in triplicate. Chemiluminescence signal detected as relative luminescent units (RLU) indicates SHP1 or SHP2 recruitment to PD-1. EA Enzyme acceptor, ED enzyme donor. **B** Representative dose–response curve for SHP1 and SHP2 recruitment in the Jurkat PD-1 SHP1 and SHP2 signaling cell lines; **C** Graphical representation of the EC50 and EC90 values calculated from dose–response curves from 5 independent experiments. Data are shown as mean ± SEM, *p* < 0.05 considered significant

Recently, studies have shown that certain molecular features such as epitope position and binding affinity can render PD-1 monoclonal Abs to have antagonist or agonistic activity suggesting different pharmacology based on the binding epitope [26, 27]. Considering the differences in binding kinetics between toripalimab and pembrolizumab that was demonstrated above and the known differences in their binding sites on the PD-1 molecule [28], we investigated whether the two PD-1 Abs would differ in PD-1 receptor proximal signaling. The PathHunter® Jurkat PD-1 SHP1/2 Signaling Cell Lines enable estimating the strength of ligand-based activation of PD-1 by quantifying the levels of recruitment of SHP1 or SHP2 to the PD-1 receptor. Upon treating PathHunter® Jurkat PD-1 cell lines expressing either the SHP1 or SHP2 with increasing concentrations of toripalimab or pembrolizumab, a dose-dependent increase in PD-1 signaling was observed for both toripalimab and pembrolizumab. Pembrolizumab showed a higher potency than toripalimab (data not shown, EC_50_ for SHP1 were 28.80 ng/mL and 873 ng/mL; and those for SHP2 were 2.30 ng/mL and 44.80 ng/mL, for pembrolizumab and toripalimab, respectively). Subsequently, to overcome low signal intensities, signal amplification was done by cross-linking the bound PD-1 Abs using FcγRIIb expressing U20S cells in co-culture with the PathHunter® Jurkat PD-1 SHP signaling cells (Fig. 5A) [26, 27]. Consistent with the previous experiment, pembrolizumab exhibits a higher recruitment of SHP1 and SHP2 compared to toripalimab (Fig. 5B). Plotting the EC_50_ values, it was further demonstrated that pembrolizumab had ~ 10- and ~ 14-fold higher potential than toripalimab to recruit SHP1 and SHP2, respectively (Fig. 5C). Pembrolizumab also had significantly lower EC_90_ values compared to toripalimab, indicating that lower concentrations of pembrolizumab are sufficient to achieve the maximal SHP1 and SHP2 recruitment (Fig. 5C). Other FDA-approved PD-1 Abs (nivolumab and cemiplimab) were also evaluated in this experimental system and toripalimab was the least potent in PD-1 recruitment of SHP1 or SHP2 (Supplementary Fig. S3). Therefore, our data demonstrate that while both toripalimab and pembrolizumab induce some level of PD-1 receptor activity, toripalimab is significantly weaker than pembrolizumab at recruiting SHP1 and SHP2 phosphatases.

### Toripalimab induces an elevated IFN-γ signature in dissociated NSCLC tumor and immune cells

**Fig. 6:**
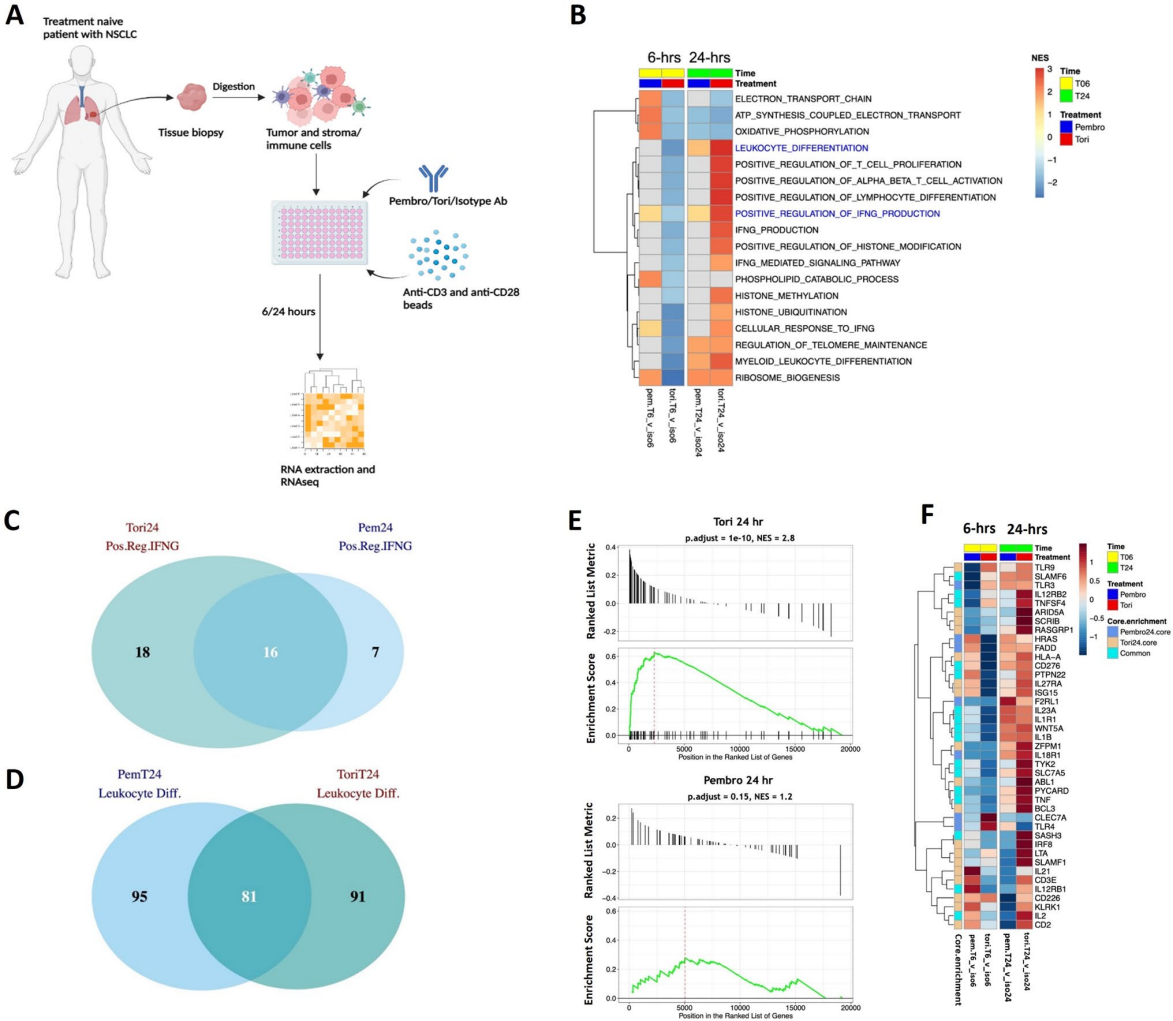
Toripalimab positively modulates genes associated with IFN-γ production in dissociated NSCLC tumor cells isolated from treatment naïve patients. **A** Schematic diagram of expression profiling of dissociated tumor cells treated with toripalimab (tori), pembrolizumab (pembro) or isotype control antibody. **B** GSEA analysis of Gene Ontology Biological Process gene sets. The normalized expression scores (NES) from pathways for each PD-1 Ab (tori or pembro) treatment compared to control at each time point are shown. Pathways were filtered with *p*.adjust < 0.05 followed by combinatorial or unique filtering by the various treatment comparisons. **C** A Venn diagram comparing the core enrichment genes from the 24 h time point for “Positive regulation of IFNG production” with the PD-1 Abs. **D** A Venn diagram comparing the core enrichment genes from the 24 h time point for “Leukocyte differentiation.” A table of these core enrichment genes is included in the Supplement Fig. S4. **E** Leading edge plots comparing “Positive regulation of IFNG production” by tori (top) and pembro (bottom). **F** Heatmap of the core enrichment genes of the “Positive regulation of IFNG production” pathway after tori and pembro treatment. Sixteen of the 41 combined core enrichment genes are common to both PD-1 Ab treatments (cyan). Seven of the core enrichment genes are unique to pembro treatment (light blue) while 18 of the core enrichment genes are unique to tori treatment (tan)

Toripalimab and pembrolizumab do not bind to murine PD-1, and thus, in vivo studies are not possible to evaluate these antibodies. We therefore utilized a model employing dissociated human tumors to evaluate immune cell activation following toripalimab or pembrolizumab ex vivo treatment, and we examined gene expression in dissociated tumor cells (DTC) that may mimic the cellular components of the tumor microenvironment. Human tumors from 16 patients with treatment naïve NSCLC were harvested, mechanically and enzymatically digested, and treated with isotype control Ab, toripalimab or pembrolizumab in the presence of anti-CD3 and anti-CD28 Abs, for 6 and 24 h (hr) (Fig. 6A). The patient demographics and tumor/immune cell sample characterization via flow cytometry analysis are provided in Supplementary Table ST2.

Upon differential gene expression analysis, treatment with either toripalimab or pembrolizumab showed 96 genes that were differentially expressed relative to the control Ab (56 upregulated and 40 downregulated) at 6 and 24 h (abs(logFC) > 1.25 and *p* < 0.05, Supplementary Table ST3). Gene Set Enrichment Analysis (GSEA) using Gene Ontology Biological Process (GOBP) showed the most significant canonical pathways and immunological networks that could differentiate the effects of toripalimab and pembrolizumab (Fig. 6B). At the 6 h time point, pembrolizumab treatment-enriched gene sets that were significantly enriched in metabolic pathways included “ATP synthesis coupled electron transport,” “Oxidative Phosphorylation,” “Positive regulation of IFNG production” and “Cellular response to IFNG.” At the 24 h time point (Fig. 6B), treatment of DTCs with either of the PD-1 Ab resulted in enrichment of gene sets associated with “Positive regulation of IFNG production” with toripalimab showing a stronger normalized expression score (NES) of 2.8 compared to NES of 1.2 for pembrolizumab. Similarly, a stronger NES was observed upon toripalimab treatment for the “Leukocyte differentiation” (3.04 and 1.46 for toripalimab and pembrolizumab, respectively) and “Myeloid differentiation” (2.5 and 1.73 for toripalimab and pembrolizumab, respectively) pathways. Pathways involved in lymphocyte activation that included “Positive regulation of T cell proliferation,” “Positive regulation of alpha–beta T cell activation” and “Positive regulation of lymphocyte differentiation” and “Leukocyte differentiation” were highly upregulated in response to toripalimab treatment (Fig. 6B). Additionally, we also observed enrichment of gene sets associated with macrophage activation, polarization and migration upon treatment with toripalimab at both 6 h and 24 h, in comparison with pembrolizumab (Supplementary Fig. S5).

Of the different gene sets, based on the known mechanism of action of PD-1 Abs we looked closely at the “Positive regulation of IFNG production” and “Leukocyte differentiation” gene sets, which are most relevant. Venn diagram analysis showed that in the “Positive regulation of IFNG production” gene set, at the 24 h time point, 18 core enrichment genes were unique for toripalimab, 7 for pembrolizumab and 16 were common between both PD-1 Abs (Fig. 6C). On the other hand, for the “Leukocyte differentiation” gene set, 95 were unique to toripalimab, 91 to pembrolizumab and 81 were in common between both PD-1 Abs (Fig. 6D). These findings indicate that toripalimab more positively modulates genes associated with IFNG production than pembrolizumab. Furthermore, leading edge enrichment plots of the “Positive regulation of IFNG production” gene set showed a more asymmetric profile and increased NES score with toripalimab treated samples in comparison with pembrolizumab (Fig. 6E). A heatmap of the 41 core enrichment genes modulated by toripalimab in this gene set are shown in Fig. 6F. At 6 h, only two genes (CLEC7A and TLR4) were differentially elevated by toripalimab, while 13 genes were differentially elevated by pembrolizumab (HRAS, FADD, HLA-A, CD276, PTPN22, IL27RA, ISG15, IL21, CD3E, IL12RB1, KLRK1, IL2 and CD2). However, at 24 h, 17 genes were elevated by toripalimab. These include: IL12RB2, TNFSF4, ARID5A, SCRIB, PTPN22, TYK2, ABL1, SASH3, IRF8, LTA, SLAMF1, CD3E, IL12RB1, CD226, KLRK1, IL2 and CD2.

**Fig. 7:**
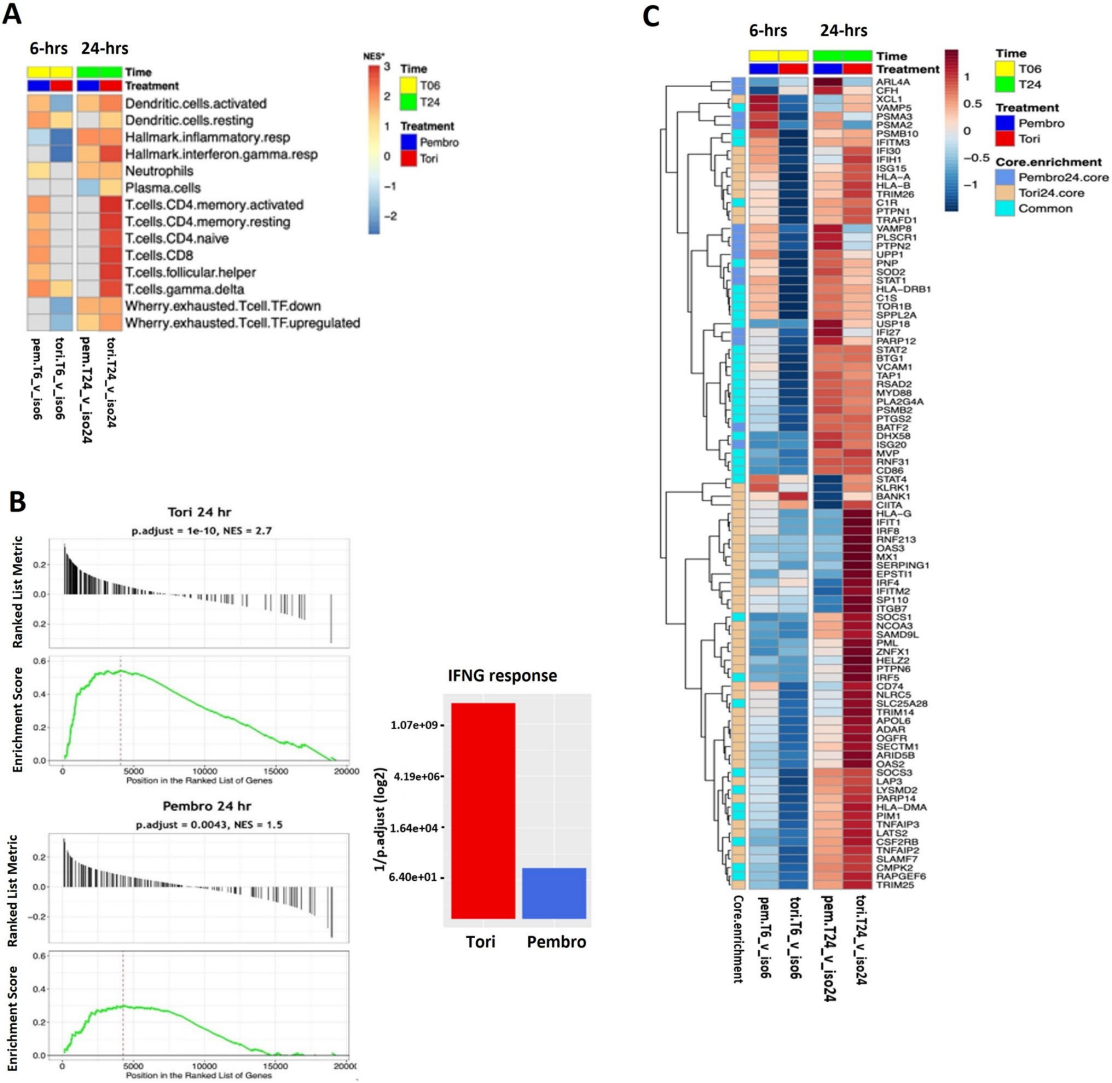
Toripalimab induces an elevated IFN-γ gene signature in dissociated NSCLC tumor cells isolated from treatment naïve patients. **A** Custom immune cell and interferon response pathway analysis. All NES scores were filtered by an exploratory *p*.adjust < 0.15. **B** Leading edge plots of “Hallmark interferon response” following toripalimab (tori, top) or pembrolizumab (pembro) treatment at 24 h. **C** Heatmap of 94 core enrichment genes from the interferon response pathway after 6 and 24 h treatments with pembro and tori. NES, normalized expression score; *p*.adj, *p*-adjusted (Bonferroni–Hochberg); log2FC, log2-fold change; iso6, isotype at 6 h, iso24, isotype at 24 h; Core.enrichment: Core enrichment genes from the leading edge’s subset of genes from the gene set are those genes that drive the enrichment score in the GSEA analysis. T06, 6 h time point; T024, 24 h time point

We next performed pathway analysis on a custom gene set comprising immune cell subtype signatures derived from Wherry et al. [29] and the LM22 matrix derived from multiple public expression datasets of sorted immune cell subtypes published in Chen et al. [30]. To bridge and complement the GOBP analysis, we also included two Hallmark gene sets to represent IFN-γ response and inflammatory response. At 6 h, pembrolizumab treatment uniquely resulted in elevation of gene sets belonging to dendritic cell pathways, neutrophil pathway and T cell pathways (Fig. 7A). At 24 h, toripalimab treatment elevated several important CD4 and C8 gene sets and the “Hallmark IFNG response” gene set. Additionally, it is important to note that some gene sets were similarly elevated by both PD-1 Abs, such as “Hallmark inflammatory response,” “neutrophils” and “exhausted T cells.TF down.”

Similar to the GOBP analysis, the signal from the “Hallmark IFNG response” pathway was stronger in response to toripalimab treatment than to pembrolizumab at 24 h, with a more significant *P*.adjust and NES score for toripalimab (*P*.adjust: 1e-10, NES: 2.7) than pembrolizumab (*P*.adjust: 0.0043, NES: 1.5) (Fig. 7B). An analysis of the core enrichment genes from the “Hallmark IFNG response” pathway at 6 h showed differential elevation of multiple IFN-γ response genes with pembrolizumab, but hardly any with toripalimab. However, at 24 h, toripalimab treatment elevated several important genes compared to pembrolizumab (Fig. 7C). Here, the genes that account for the “Hallmark IFNG response” are downstream of IFN-γ signaling, which is different from the “positive regulation of IFNG production” gene set analyzed above, which includes regulatory genes upstream of IFN-γ production. The lack of overlap between the two gene sets is confirmed as we only find 4 genes (HLA-A, IRF8, ISG15 and KLRK1) that were in common between the core enrichment genes from the two gene sets (Supplementary Fig. S4) indicating that the core enrichment genes upregulated were unique to each antibody. Additionally, treatment of DTCs with toripalimab resulted in activation of myeloid cells as observed by the enrichment of gene sets associated with macrophage activation (Fig. 6B and Supplementary Fig. S5).

In summary, our data demonstrate that in an ex vivo system consisting of dissociated and stimulated human NSCLC tumors, toripalimab elevated the expression of IFN-γ related gene signature, compared to pembrolizumab, consistent with our in vitro stimulated T cell experimental findings.

## Discussion

The results presented here demonstrate the molecular and functional characteristics of toripalimab and the post hoc clinical observation of toripalimab in combination with chemotherapy. Antibody affinity is one of the key properties affecting the potency of therapeutic antibodies, and upon comparing binding affinities, toripalimab demonstrates a higher binding affinity with a slower dissociation rate constant (off-rate) than pembrolizumab. It has been shown that higher binding affinity improves the potency of antagonistic antibodies in blocking ligand binding and cellular signaling [31]. In addition to its higher binding affinity to PD-1, toripalimab is distinguished from pembrolizumab in its distinct binding sites on PD-1 at the FG loop [28]. The FG loop of PD-1 is a crucial binding site for its interactions with PD-L1 and is a “hot spot” for PD-1/PD-L1 blockade [32]. Stable synapse formation at the T cell–APC conjugation is required for T cell activation and is regulated by the accumulation of PD-1 at the immunological synapse [33, 34]. We hypothesize, for further studies to continue to explore, that the unique epitope binding, higher binding affinity and slower dissociation rate of toripalimab, may facilitate a prolonged inhibition of the PD-1 interaction with its ligands, thereby preventing its accumulation at the synapse leading to greater T cell activation.

Based on the differences in epitope and binding kinetics that we observed between toripalimab and pembrolizumab, we expected the two PD-1 Abs to have similarities and differential ability to activate T cells. In fact, an increased potency of toripalimab in enhancing T cell activation was demonstrated in multiple in vitro functional assay systems using primary human T cells. Importantly, both toripalimab and pembrolizumab treatment result in T cell activation confirming the known mechanism of action. In a TCR activation-dependent system, SEB-stimulated PBMCs demonstrated increased levels of IFN-γ and IL-2 in the presence of both toripalimab and pembrolizumab indicating both Abs were able to activate T cells upon checkpoint blockade. However, toripalimab induced higher expression of IFN-γ, IL-2, TNF, GM-CSF and IL-18 indicating a stronger Th1 response compared to that elicited by pembrolizumab. IFN-γ plays a major role in anticancer immunity by upregulating MHC expression and antigen presentation by dendritic cells, and additionally activating cytotoxic T cells, NK cells and tumoricidal M1 macrophages [35, 36].

The selective upregulation of Th1 cytokines over the Th2 cytokines and TH17 cytokines by toripalimab, in comparison with pembrolizumab, implies a differential regulation of cytokine responses between the two Abs upon binding to PD-1. The significant upregulation of myeloid-derived inflammatory cytokines IL-1α and IL-1β that we observe may be possibly explained by the pleiotropic activation of myeloid cells by the toripalimab responsive Th1 cells in our assay [37]. Interestingly, in an earlier report by Harper et al. [38] comparing inflammatory cytokine expression induced by nivolumab and pembrolizumab in an MLR assay did not demonstrate any significant differences in levels of inflammatory cytokines secreted. IL-10 is an immunoregulatory cytokine with its presence in tumors resulting in suppression of anti-tumor T cell responses and poor cancer progression [39, 40]. Though comparable levels of expression of IL-10 were observed in response to treatment with either toripalimab or pembrolizumab in our study, only treatment with toripalimab skews the ratio of inflammatory to suppressive cytokine toward an inflammatory phenotype.

Similar to SEB-stimulated PBMCs, in anti-CD3- and anti-CD28-activated human CD8 + T cells, toripalimab induced significantly increased secretion of IFN-γ in comparison with control treated cells, while no significant increase was observed with pembrolizumab. Though this assay is characterized by the absence of PD-L1 expressing APCs or cancer cells, the possibility of interaction between T cell intrinsic PD-1 and PD-L1 cannot be excluded in this in vitro system [41]. It has been demonstrated that within the TME ≈5% of CD8 T cells could express PD-L1 and engage in regulatory activities [42]. Interestingly, in this in vitro assay, upon activation of CD8 + T cells isolated from healthy human PBMCs with anti-CD3/CD28 antibodies, we observed an increase in expression of PD-L1 in addition to expression of PD-1 (Supplementary Fig. S6). This supports a possible role for T cell intrinsic PD-1 and PD-L1 expression as a mechanism for the higher activity of toripalimab in this assay. Robust assays measuring T cell signaling upon engaging PD-1/PD-L1 axis on CD8 T cells will be better suited to further investigate and understand this mechanism, and its impact on in anti-PD-1 check point blockade.

Given that these PD-1 Abs do not have murine cross-reactivity, we leveraged an ex vivo system using primary human tumors. Upon ex vivo testing of the PD-1 Abs in excised and dissociated human tumor biopsies that mimic the cellular composition of the tumor microenvironment, we observed a difference in kinetics and intensity of IFN pathway gene activation in response to treatment with toripalimab and pembrolizumab. Toripalimab significantly elevated the IFN-γ gene signature when compared to pembrolizumab at 24 h after T cell activation. Pathways associated with IFN-γ upregulation and response from the GOBP and the Hallmark gene sets were strongly upregulated at 24 h post-T cell activation compared to pembrolizumab. Pembrolizumab on the other hand upregulated pathways associated with metabolism and IFN-γ at 6 h with little or no upregulation at 24 h. Interestingly, though both Abs upregulated the IFN-γ-associated pathways, the core enrichment genes upregulated were unique to each antibody, further underscoring their distinctive mechanisms for T cell activation. Though we observe an enrichment of T cell exhaustion signature by both PD-1 abs, we believe this may not be representative of T cell exhaustion in this assay since several genes normally associated with T cell activation are also expressed by T cells at higher levels in the exhausted state. Furthermore, the 24 h time point for treatment with anti-PD-1 antibodies in this experiment may not be sufficient to drive T cell exhaustion. As a chronic IFN-γ signaling is required for T cell exhaustion [43], a chronic T cell activation model may be better suited to assess the effect of the anti-PD-1 antibodies on T cell exhaustion and will be considered in future studies. Additionally, treatment with toripalimab is potentially resulting in activation of myeloid cells as observed by upregulation of myeloid-derived cytokines in the PBMC-based assay and in the enrichment of gene sets associated with macrophage activation in DTCs. Myeloid cells have long been noted to contribute to the formation of an immunosuppressive tumor promoting niche in the TME [44] and myeloid-specific PD-1 targeting could mediate myeloid cell–intrinsic effects that initiate systemic anti-tumor responses. We hypothesize that the higher binding affinity of toripalimab with its longer dissociation rate compared to pembrolizumab could plausibly explain the time-dependent responses to the two Abs and differential gene expressions observed in this assay. It is known that antibodies with long *k*_d_ can take many hours to reach equilibrium [45]. Additionally, differences in gene expression profiles may also be explained by the binding of toripalimab and pembrolizumab to different epitopes on PD-1. Further experiments are required to evaluate this.

Antagonistic Abs commonly have partial agonistic properties upon binding to their target receptor [27]. We show that several PD-1 Abs have these properties, however pembrolizumab demonstrated higher partial agonistic potential by activating the PD-1 receptor and recruiting SHP1 and SHP2 phosphatases than toripalimab. Importantly, the ability of pembrolizumab to activate the PD-1 receptor was amplified by FcγRIIB-mediated cross-linking. The extent of antagonist and agonist functions of blocking PD-1 Abs is determined by their epitope specificity and binding affinities [26, 46, 47]. As mentioned earlier, toripalimab is distinguished from pembrolizumab in its distinct binding sites on PD-1 in that the FG loop of PD-1 is a crucial binding site for its interactions with PD-L1 [32]. Upon binding to PD-1, toripalimab blocks PD-L1 binding by competing for the FG loop and sterically hindering the binding of PD-L1 [28]. In contrast, pembrolizumab binds to PD-1 mainly through its C′D loop but requires the interactions with residues in the C, C′ and F strands, and the BC and FG loops of PD-1 to compete with the binding of PD-L1 and block the PD-1/PD-L1 interaction [48, 49]. Toripalimab’s unique binding epitope on PD-1 and higher binding affinity may contribute to its weak PD-1 receptor activation compared to pembrolizumab. Studies evaluating the differences in downstream signaling by the two Abs are currently being performed to further elucidate their differences in immune activation.

Taken together, our results from our in vitro and ex vivo studies demonstrate the ability of toripalimab as an ICI to induce a strong T cell response dominated by an IFN-γ signature that could contribute to enhanced anti-tumor immune responses. In the recent clinical data, we present here and previously published [50–53], toripalimab in combination with chemotherapy conferred an overall survival benefit over chemotherapy alone in NPC, NSCLC and ESCC patients, irrespective of their PD-L1 status in subgroup analyses. Additionally, no new PD-1-associated adverse events were reported in these studies. In the NPC study (JUPITER-02), the hazard ratio for the toripalimab plus chemotherapy compared to chemotherapy alone group indicated that toripalimab plus chemotherapy reduced the risk of death for both TPS ≥ 1% and TPS < 1% subgroups with TPS < 1% subgroup showing greater benefits. Importantly, we observed in the NSCLC study that toripalimab plus chemotherapy treatment resulted in similar benefits in survival in both TPS ≥ 1% and TPS < 1% subgroups. Unlike TPS that counts the PD-L1 positive tumor cells, CPS takes into count the immune cells expressing PD-L1 within the tumors. Comparing the OS of ESCC patients with a CPS ≥ 1 with CPS < 1, similar improvements in OS were observed in patients treated with toripalimab plus chemotherapy belonging to both subgroups. This contrasts with conclusions from a meta-analysis performed by Noori et al. [54] on results from randomized clinical trials treating patients suffering from esophageal carcinoma with PD-1/PD-L1 inhibitors, which showed that only a CPS = 10 was predictive of a lower rate of mortality when PD-1 inhibitors including pembrolizumab and nivolumab were administered. Similarly, the results from the KENOTE-590, CheckMate 648 and ESCORT-1st clinical trials suggest a positive correlation between the PD-L1 expression level and the efficacy of PD-1 blockade plus chemotherapy in esophageal cancer [55–57].

It is important to note that clinical studies evaluating toripalimab as monotherapy treatment result in better clinical activity in patients with PD-L1 positive tumors compared to PD-L1 low tumors [58]. We speculate the activity, irrespective of PD-L1 status, of toripalimab with chemotherapy may be explained by higher T cell activation, and in inflamed tumors, the presence of chemotherapy potentiates the immune response by stimulating antigen release [59] resulting in better clinical outcomes. Additional studies are required to evaluate this further particularly in clinical samples. While these observations for toripalimab are noted, limitations of these post hoc analyses include being retrospective, not statistically powered, patient demographics being solely from Asia and using a different PD-L1 assay[60]. Future clinical studies are needed to explore the activity of toripalimab in combination with chemotherapy in relation to PD-L1 status in immune responsive and nonresponsive tumors. As several PD-1 Abs are in clinical development and approved, it may be important to characterize the different antibodies pharmacology to not assume all PD-1 Abs are the same.

In summary, results from this study demonstrate that toripalimab is a potent anti-PD-1 Ab demonstrating clinical benefits when used with chemotherapy in patients irrespective of their PD-L1 status. Designed to bind to a unique epitope, and with higher affinity for PD-1, toripalimab is potent in inducing an inflammatory immune response that could augment anti-tumor responses in vivo. These characteristics of toripalimab present it as a next generation PD-1 checkpoint inhibitor. Future prospective multiregional clinical trials evaluating the efficacy of toripalimab with chemotherapy in PD-L1 subgroups warrant further evaluation.

## Materials and methods

### Antibody reagents

Pembrolizumab (Merck & Co., lot T034261), toripalimab (Junshi Biosciences, lot 202007024) and control antibody (Ab) human IgG4 (SinoBiological, Cat No. 13505-HNAH) were stored in − 80 °C in smaller aliquots to avoid repeated freeze thaw cycles.

### Study design and participants

JUPITER-02 is a published multicenter, randomized, double-blind, placebo-controlled, phase 3 trial evaluating the efficacy and safety of toripalimab plus gemcitabine and cisplatin (chemotherapy) versus placebo plus gemcitabine and cisplatin as the first-line treatment for patients with recurrent or metastatic NPC in China, Taiwan and Singapore [61] (ClinicalTrials.gov identifier: NCT03581786).

CHOICE-01 is a published multicenter, randomized, double-blind, placebo-controlled, phase 3 trial evaluating the efficacy and safety of toripalimab plus chemotherapy versus placebo plus chemotherapy as the first-line treatment for patients with NSCLC in China [16] (ClinicalTrials.gov identifier: NCT03856411). Chemotherapy regimen for nonsquamous NSCLC patients was pemetrexed plus cisplatin or carboplatin and that for squamous NSCLC patients was nab-paclitaxel plus carboplatin.

JUPITER-06 is a published multicenter, randomized, double-blind, placebo-controlled, phase 3 trial evaluating the efficacy and safety of toripalimab plus paclitaxel and cisplatin (chemotherapy) versus placebo plus paclitaxel and cisplatin as the first-line treatment for patients with advanced ESCC in China [60] (ClinicalTrials.gov identifier: NCT03829969).

### Outcomes

The end point used for this post hoc analysis was OS according to PD-L1 subgroups using patient-level data as of November 18, 2022 (JUPITER-02) [62], October 21, 2021 (CHOICE-01) [16] and March 22, 2021 (JUPITER-06). In JUPITER-02, CHOICE-01 and JUPITER-06, PD-L1 expression in archival or fresh tumor biopsy samples obtained from patients before treatment was stained and scored centrally in a blinded manner using the JS311 antibody and a validated staining assay. A cross-correlation study showed concordance between JS311 and PD-L1 antibodies used in diagnostic tests including 22C3, SP263 and 28-8 antibodies [63]. PD-L1 TPS was defined as the percentage of viable tumor cells with partial or complete membrane staining of PD-L1 in at least 100 viable tumors. CPS PD-L1 positivity was defined as the presence of membrane staining of any intensity in ≥ 1% of tumor cells or ≥ 1% of immune cells.

### T cell activation measured by cytokine secretion assay with SEB-stimulated human PBMCs

Healthy human PBMCs (Stemcell Technologies, Cat No. 70025.1) were thawed, washed and plated at 0.1 or 0.5 million cells/well in a total volume of 100 µL in media (RPMI-1640, 10% FBS) in 96-well U-bottomed plates. PBMCs are stimulated in triplicate with 100 ng/mL SEB (Sigma, Cat No. 324798). Pembrolizumab, toripalimab or control Ab were added to the PBMCs at the time of stimulation at concentrations of 10, 3.3 or 1.1 μg/mL. After three days incubation in a 37 °C, 5% CO_2_ incubator, supernatants were collected for analysis of IFN-γ and IL-2 expression by ELISA (R&D Systems, Cat No. DY285B and DY202) following manufacturer’s protocol using a SpectraMax iD3 (Molecular Devices).

### T cell activation measured by cytokine secretion assay with CD3/CD28-stimulated human naïve CD8 + T cells

Frozen peripheral blood naïve CD8 + T cells from human healthy donors (STEMCELL Technologies, Cat No. 70027) were thawed and rested in Immunocult-XF T cell expansion media (STEMCELL Technologies, Cat No. 10981) for 2–3 h in 37 °C, 5% CO_2_ incubator. 96-well flat-bottomed plates were coated with 0.5 μg/mL antihuman CD3 (Biolegend, Cat No. 300465)), 0.5 μg/mL antihuman CD28 (Biolegend, Cat No. 302948) and 10 μg/mL Ab (pembrolizumab, toripalimab or control). After 2 h of incubation at 37 °C, the plates were washed twice with PBS and 0.2 million rested CD8 T cells per well was added and placed in a 37 °C, 5% CO_2_ incubator. After three days, cell culture supernatants were collected and tested for levels of human IFN-γ following manufacturer’s protocol (R&D Systems, Cat No. DY285B) using a SpectraMax iD3 (Molecular Devices). Following the flow cytometry analysis, CD8 T cells before and after 3 days of activation were stained with LIVE/DEAD Fixable Aqua Dead Cell Stain Kit (Invitrogen) according to manufacturer’s instructions. Cells were then washed and labeled with fluorochrome conjugated antibodies, namely, Pacific Blue antihuman CD8 antibody (Biolegend), PE/Cyanine 7 antihuman PD-L1 (Biolegend) and PE-CF594 antihuman PD-1 (BD Biosciences), using manufacturer’s instruction. Stained cells were acquired on a 3-laser Cytek Northern Lights flow cytometer (Cytek Biosciences) and analyzed using Flowjo (version 10.7.2 for Windows).

### Jurkat PD-1-SHP1 or SHP2 recruitment reporter gene assay

PathHunter® U2OS FcγRIIb cells (DiscoverX, Cat No. 93-1133C3) were seeded into an assay plate with CP0 assay medium, followed by addition of serial dilutions (0.01–10 µg/mL) of pembrolizumab, toripalimab or control Ab. The cells were incubated at 37 °C in 5% C0_2_ incubator for 1 h to allow of antibody opsonization. 4 × 10^4^ PathHunter® Jurkat PD-1 (SHP1) or PD1 (SHP2) signaling cells (DiscoverX, Cat. No 93-1104C19 and 93-1106C19) were then added per well to the antibody-opsonized PathHunter® U2OS FcγRIIb cells and incubated for 2 h at room temperature. The assay plate was processed using the PathHunter Bioassay Detection Kit (DiscoverX, 93-0933), and the detection of assay signal was evaluated on a PerkinElmer Envision (0.2 s integration time). Data were plotted using GraphPad Prism 8.3; EC50 values were calculated using a sigmoidal dose–response curve fit with variable slope (four parameter) with no constraints and EC90 values with a constraint *F* = 90; fit method = least squares (normal fit).

### Antibody binding affinity and kinetics

Surface plasmon resonance (SPR) experiments were performed using a Biacore T200 optical biosensor (Cytiva Life Sciences). The PD-1 protein used in the SPR studies was a his-tagged monomeric protein (deglycosylated MW = 16800 and glycosylated MW of 28620) and was purchased from Acro Biosystems (Cat No. PD1-H5221)). The active PD-1 concentration was determined by Calibration-Free Concentration Analysis (CFCA) on a Biacore T200 instrument using a MW of 28,620 and a diffusion coefficient (20 °C) of 8.84 × 10^–11^ m^2^/s[64]. All PD-1 samples were prepared in vacuum-degassed HBS-P + buffer (0.01 M Hepes, 0.15 M NaCl, 0.05% surfactant P-20) from Cytiva Life Sciences with 100 µg/mL filtered BSA. C1 sensor chips (Cytiva Life Sciences, Cat No. BR-1005-35), amine-coupling reagents (Cytiva Life Sciences , Cat No. BR-1000–50), 1-ethyl-3-(3-dimethylaminopropyl) carbodiimide (EDC), N-hydroxysuccinimide (NHS), ethanolamine, 10 mM sodium acetate buffer pH 5.0 (Cytiva Life Sciences, Cat. No BR-1003-51) and 10 mM glycine–HCl pH 2.0 and 2.5 were purchased from Cytiva Life Sciences (BR-1003-55 and BR-1003-56).

For Biacore kinetic measurements, standard EDC/NHS coupling was used to covalently immobilize toripalimab and pembrolizumab to three flow cells on two C1 sensor chips. C1 chips were activated with EDC/NHS for 5 min with excess activated carboxyl groups blocked with ethanolamine for 5 min following immobilization of mAb (diluted to 85 µg/mL in 10 mM sodium acetate, pH 5.0) to three flow cells. Immobilization levels for toripalimab and pembrolizumab over the three flow cells ranged from 577 to 643 RU and 425 to 455 RU, respectively. For all experiments, one flow cell served as a reference surface following activation and blocking on each chip in the absence of mAb immobilization. All Biacore kinetic experiments were conducted at 25 °C. All flow rates used in the kinetic experiments were 100 mL/min.

For the PD-1/toripalimab kinetic experiment, seven antigen concentrations (twofold serial dilutions) were prepared in running buffer and injected in triplicate in a random order for 3 min at 100 µL/min followed by 5 min of dissociation data with buffer injections every sixth injection for double-referencing. To obtain more extensive dissociation decay data, each experiment included 3 additional injections of the highest antigen concentration alternated with 3 additional buffer injections followed by a dissociation phase of 90 min. The surfaces were regenerated with three 15-s pulses of 10 mM glycine-HCl, pH 2.0. PD-1 was injected randomly in triplicate for one minute at six antigen concentrations (twofold serial dilutions) over three pembrolizumab-immobilized flow cells with buffer injections every sixth injection. The dissociation phases were followed for 3 min. Regeneration was achieved with three 30 s pulses of glycine-HCl, pH 2.5.

Biacore sensorgrams were processed and fit using Scrubber software (version 2.4.0.8, BioLogic Software). Sensorgrams were first zeroed on the y axis and then x-aligned at the beginning of the Ag injection. Bulk refractive index changes were removed by subtracting the responses from the reference flow cell from the experimental flow cells. The average or closest response of all blank injections was subtracted from all PD-1 sensorgrams to remove systematic artifacts in the experimental flow cells. Sensorgram data from each flow cell were globally fit to a 1:1 bimolecular interaction model with a term for mass transport included to calculate *k*_a_ and *k*_d_. The *K*_D_ was calculated from the quotient of *k*_d_/*k*_a_.

### DTC cell culture

Sixteen NSCLC adenocarcinoma lung cancer dissociated tumor cell (DTC) samples were thawed at 37 °C, diluted in an equal volume of T Cell Culture Media (Immunocult-XF T Cell Expansion Media + 1X Penicillin/Streptomycin/L-Glutamine + 4 mg/mL Amphotericin B + 50 mg/mL Gentamycin) and counted using acridine orange/propidium iodide on a Nexcelom Cellometer. The cells were pelleted, resuspended in T Cell Culture Media and incubated at 37 °C, 5% CO_2_ for 30 min. After 30 min, 500,000 cells/well were plated into a 96-well round-bottomed plate, at 3 wells per plate for each time point.

The samples were treated for the times indicated, and RNA was isolated from each lung cancer dissociated tumor cell. For one well on each plate, pembrolizumab, toripalimab or isotype control antibody was added to a final concentration of 10 mg/mL. The cells from all sample plates were incubated for 15 min at 37 °C, 5% CO_2_. Following this incubation, anti-CD3 (Biolegend, Cat No. 317326) and anti-CD28 (Biolegend, Cat No. 302934) antibodies were added at a final concentration of 2 ug/ml and cells were cultured for another 5 min at 37 °C, 5% CO_2_ prior to the addition of anti-mouse IgG (Invitrogen, Cat No. A16068) at a final concentration of 10 ug/ml and incubated for another 6 h or 24 h at 37 °C, 5% CO_2_. At each time point, the cells were harvested for RNA isolation and the supernatant was removed for cytokine analysis.

### RNA isolation, library preparation and sequencing

For RNA isolation, cells were pelleted and lysed in Buffer RLT containing b-mercaptoethanol.

RNA was processed using the Qiagen RNeasy Plus Micro kit (Cat No. 74034) using QIAShredders, and RNA quality and quantity were assessed on an Agilent TapeStation 4150 using the High Sensitivity RNA ScreenTape (Cat No. 5067-5581). The cDNA libraries from the RNA were sequenced by Discovery Life Sciences (DLS), Goleta, California. The concentration of the extracted RNAs were quantified using the fluorometric Ribogreen assay (Invitrogen, Cat No. R11490). The integrity of the extracted RNA samples was assessed for RNA Integrity Number (RIN) and DV200 score using the Fragment Analyzer (Agilent 5300). cDNA libraries were constructued using the Takara SMARTer Stranded Total RNAseq Pico Mammalian RNA amplification and library construction kit (Cat No. 634419). Libraries underwent quality control and for the libraries passing QC, RNASeq were performed on an Illumina NovaSeq6000, distributing across S4 200 single lane flow cells for 50 M 100 bp paired-end reads to reach 500 Gb sequencing. Samples that did not reach number of read or Gb specifications were topped off for additional sequencing until number of reads or Gb specifications were met.

### RNA sequencing and bioinformatics analysis

RNASeq counts were generated from paired-end sequencing following alignment/mapping to the human genome (hg38) with STAR (Dobin et al., Bioinformatics, 2013:29(1):15–21). Gene-based counts and Transcripts per Kilobase Million (TPM) were also generated and normalized with RSEM (Li et al., BMC Bioinformatics 2011: 12:323). Differential gene expression was evaluated using edgeR version 3.36.0 [64]. Plots were generated with ggplot2 version 3.4 [65], https://cloud.r-project.org/web/packages/ggplot2/index.html) within R-4.0.2 (https://cran.r-project.org). Due to the analysis of DTC samples generated in two groups, batch correction was performed with combat-seq (Zhang et al., NAR Genomics and Bioinformatics, 2020: 2(3):lqaa078) after iterative testing of gene and sample filtering parameters using principal components analysis with the preprocessCore version 1.56 R package (Bolstad B 2023, https://github.com/bmbolstad/preprocessCore).

### Pathway analysis

The logFC values from edgeR differential analysis were used for gene set enrichment analysis (GSEA). GSEA was performed with these gene sets via the clusterProfiler R version 4 package (https://bioconductor.org/packages/release/bioc/html/clusterProfiler.html, [66]. The Gene Ontology Biological Process (GOBP) and Hallmark gene sets were obtained from the Broad Institute’s msig.db website (http://www.gsea-msigdb.org/gsea/msigdb). Immune gene sets were extracted from the LM22 matrix [30] by sorting the coefficients for each of the 22 immune cell types and retaining the top 30 genes for each. Immune exhaustion gene sets were derived from the seminal paper on immune exhaustion in the LCMV model system [29].

### Statistics

Statistical tests were carried out using GraphPad Prism Software version 9. Statistical tests were used as indicated in the figure legends. *p* values are reported as follows: *p < 0.05, ** *p*< 0.01, ***p < 0.001 and ****p < 0.0001.

### Supplementary Information

Below is the link to the electronic supplementary material.

Supplementary file1 (DOCX 1524 KB)

Supplementary file2 (XLSX 3369 KB)

## Data Availability

The data supporting the findings in this study are included within the article and its supplementary materials. Raw data that support the findings will be provided upon request.
