# Supplementary material for: Toripalimab, a therapeutic monoclonal anti-PD-1 antibody with high binding affinity to PD-1 and enhanced potency to activate human T cells
Source: Cancer Immunol Immunother. 2024 Feb 24;73(3):60. doi: 10.1007/s00262-024-03635-3 (PMC10894093; doi:10.1007/s00262-024-03635-3)
Supplement: Supplementary file 1 — Supplementary file1 (DOCX 1524 KB) [file 262_2024_3635_MOESM1_ESM.docx]

**Supplementary figure S1**

**A**

**B**

**Supplementary figure S1** Sensorgrams of PD-1 binding to covalently immobilized (A) toripalimab and (B) pembrolizumab on two flow cells of a C1 chip. PD-1 was injected in triplicate for (A) 3 min in a range from 0.93-59.5 nM with dissociation followed for 5 min (top row) and dissociation for the highest concentration followed for 90 min (bottom row). PD-1 was injected in triplicate for (B) 1 min in a range from 0.63-20.3 nM with dissociation followed for 3 min. All sensorgrams were globally fit (red lines) to a 1:1 interaction model including a term for mass transport for each flow cell resulting in the rate constants and equilibrium dissociation constants shown in the inset boxes.

**Supplementary figure S2**

**
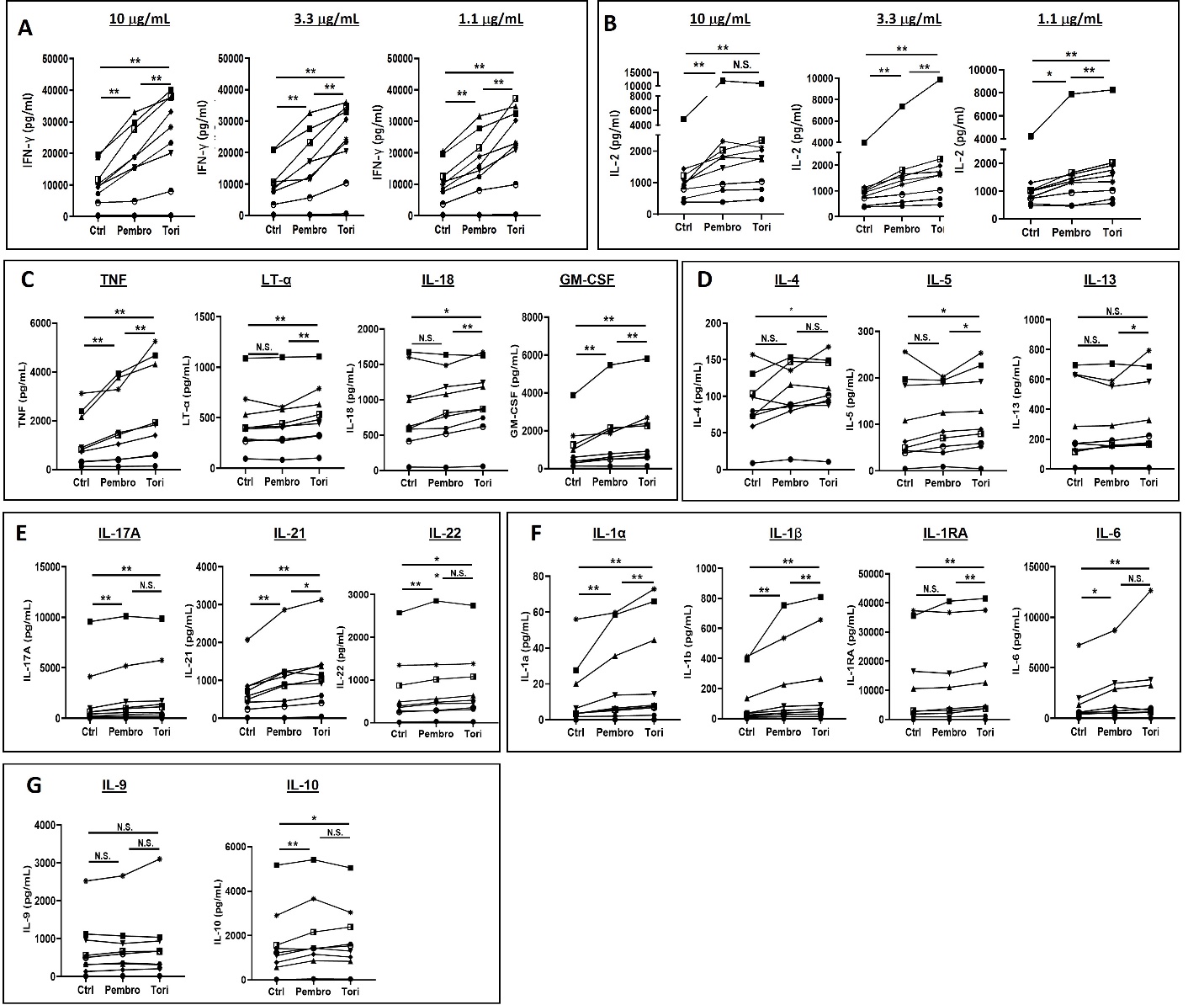
**

**Supplementary figure S2** PBMCs from nine healthy donors were cultured with 100 ng/mL SEB in the presence of 10, 3.3 or 1.1 µg/mL anti-PD-1 antibody (Ab): pembrolizumab (pembro) or toripalimab (tori); or isotype Ab control (Ctrl) in triplicate. After 3 days, cell culture supernatants were tested for levels of IFN-γ (A) and IL-2 (B) by ELISA. Supernatants collected from the 3.3 µg/mL Ab dose were quantified for levels of Th1 cytokines (C), Th2 cytokines (D), Th17 cytokines (E), myeloid derived cytokines (F), IL-9 and IL-10 (G). Each line represents one individual donor. Wilcoxon matched-pairs signed rank test was used for the statistical analysis. **P* < 0.05; ** *P* < 0.01; *** *P* < 0.001. N.S. indicates no significant difference.

**Supplementary figure S3**

**Supplementary figure S3** PathHunter® Jurkat PD-1 cell lines expressing the SHP1 or SHP2 signaling assay system were treated with increasing doses of isotype Ab control (Ctrl), pembrolizumab (Pembro), toripalimab (Tori), cemipilimab (Cemi) or nivolumab (Nivo) (dose range 0.001- 10 µg/mL) in triplicate. Chemiluminescence signal detected as relative luminescent units (RLU) indicates SHP1 or SHP2 recruitment to PD-1. Representative dose response curve from two experiments for SHP1 and SHP2 recruitment in the Jurkat-PD-1 SHP1 and SHP2 signaling cell lines. Table shows the EC50 values calculated from the dose response curves from two independent experiments.

**Supplementary figure S4**

**
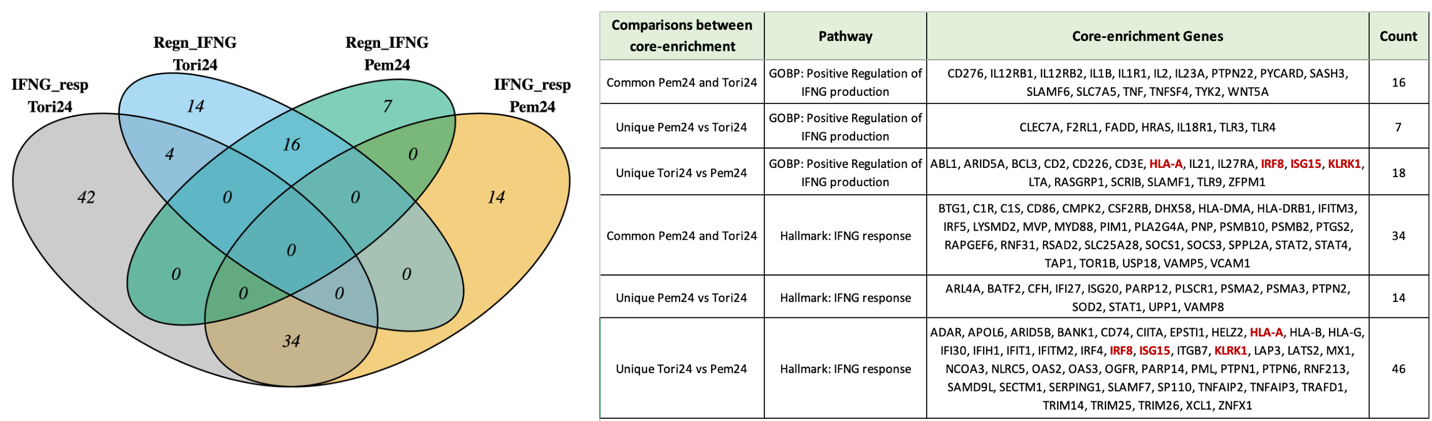
**

**Supplementary figure S4** Comparison of core-enrichment genes from “Hallmark IFNG response” and GOBP "Positive regulation of IFNG production”. The core-enrichments at 24 hours after PD-1 Ab treatments were collected and compared in a Venn diagram. The table lists the individual genes in each sector.

**Supplementary figure S5**


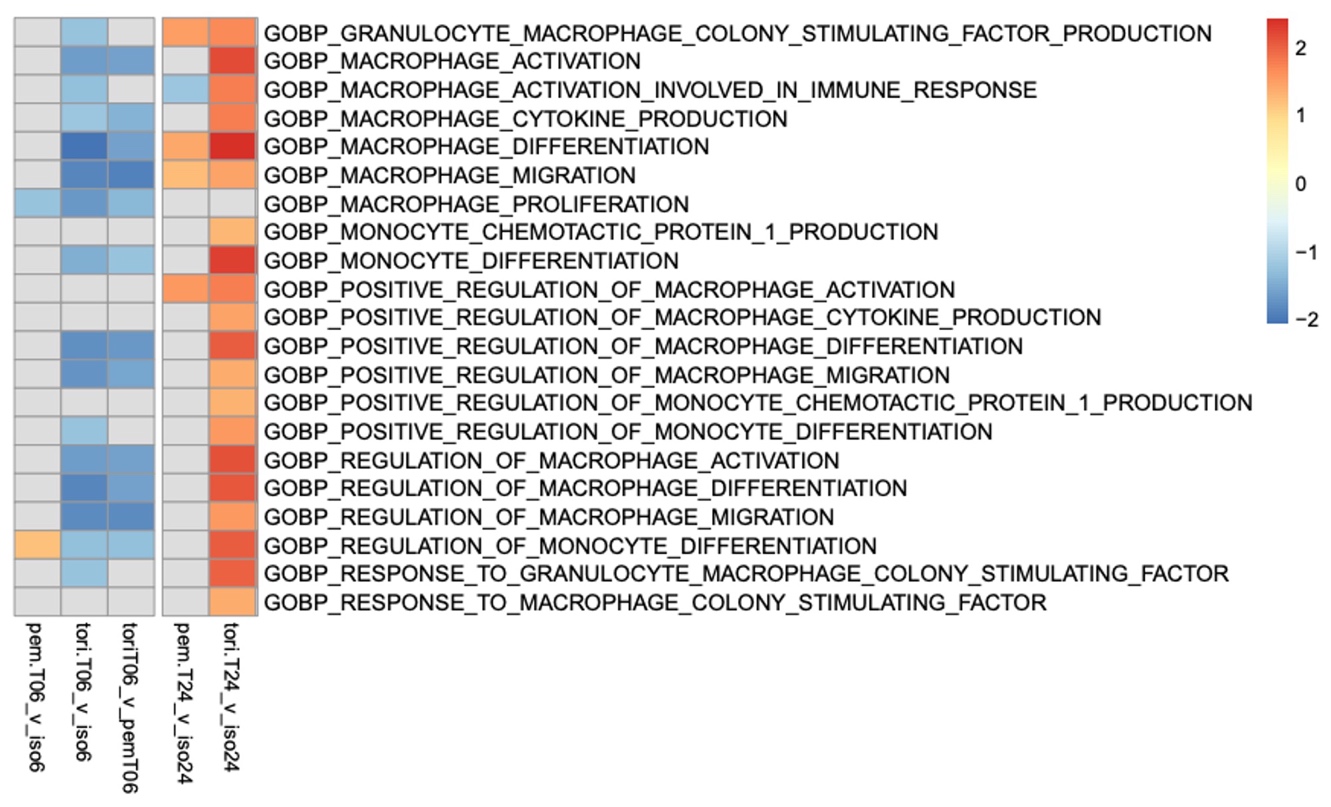


GSEA analysis of Gene Ontology Biological Process genesets for macrophage molecular signatures. The normalized expression scores (NES) from pathways for each PD-1 Ab (tori or pembro) treatment compared to control at each timepoint are shown. Pathways were filtered with p.adjust < 0.05 followed by combinatorial or unique filtering by the various treatment comparisons.

**Supplementary figure S6**


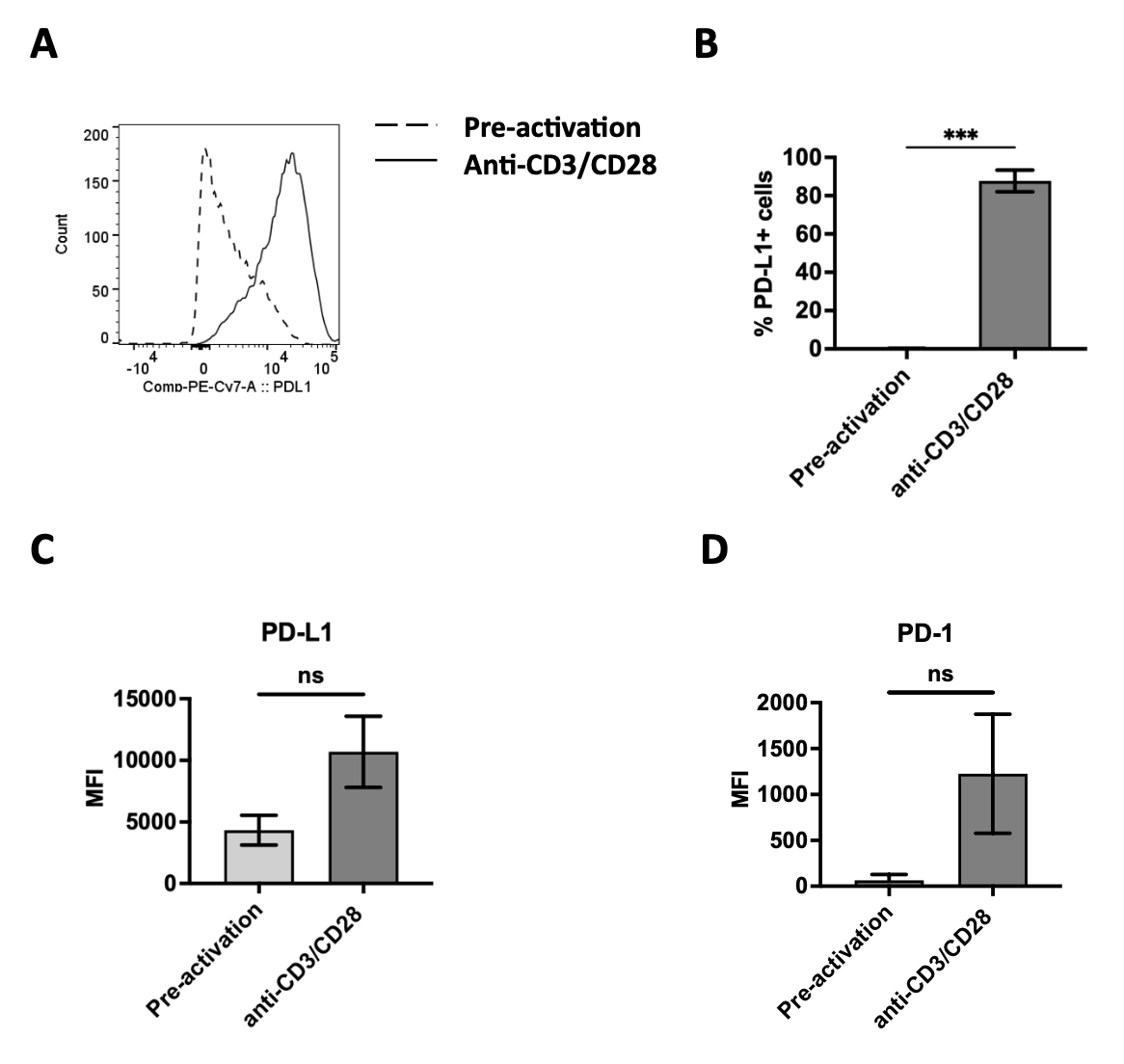


**Supplementary figure S6 CD8^+^ T cells have upregulated expression of PD-L1 after anti-CD3/CD28 activation.** Naïve CD8^+^T cells from four healthy donors were activated with human anti-CD3 (0.5 µg/mL) and human anti-CD28 (0.5 µg/mL) immobilized on a plate surface as described in “Materials and methods”. Cells were harvested before (Pre-activation) and after activation on day 3 (Anti-CD3/CD28) and expression of PD-L1 and PD-1 on the surface was analyzed by flowcytometry. (A) Representative histogram showing PD-L1 expression on CD8^+^T cells. (B) Percentage of PD-L1 expressing CD8^+^T cells. (C) PD-L1 expression on CD8^+^T cells presented as mean fluorescent intensity. (D) PD-1 expression on CD8^+^T cells presented as mean fluorescent intensity. Significant differences were calculated using paired t test. *p<0.05, ***p<0.001, ns-not significant. Data are shown as mean ± SEM.

**Supplementary Table ST1**

|  | **Average fold change in IL-2 secretion** | |
| --- | --- | --- |
| Conc. of anti-PD-1 mAb | pembrolizumab  mean±SEM | toripalimab  mean±SEM |
| 10 μg/mL | 1.66±0.53 | 1.72±0.38 |
| 3.3 μg/mL | 1.49±0.24 | 1.81±0.37 |
| 1.1 μg/mL | 1.37±0.32 | 1.59±0.29 |

PBMCs from nine healthy donors were cultured with 100 ng/mL SEB in the presence of 10, 3.3 or 1.1 µg/mL anti-PD-1 antibody (Ab): pembrolizumab or toripalimab or isotype Ab control (Ctrl) in triplicate. After 3 days, cell supernatants were collected to examine IL-2 levels by ELISA. Average fold change (mean ± SEM, n = 9) in IL-2 secretion in the presence of pembrolizumab or toripalimab relative to Ctrl at the three different concentrations of treatment are shown.

**Supplementary Table ST2** Demographics & tumor sample characterization of dissociated tumor cells harvested from patients with treatment naïve NSCLC.


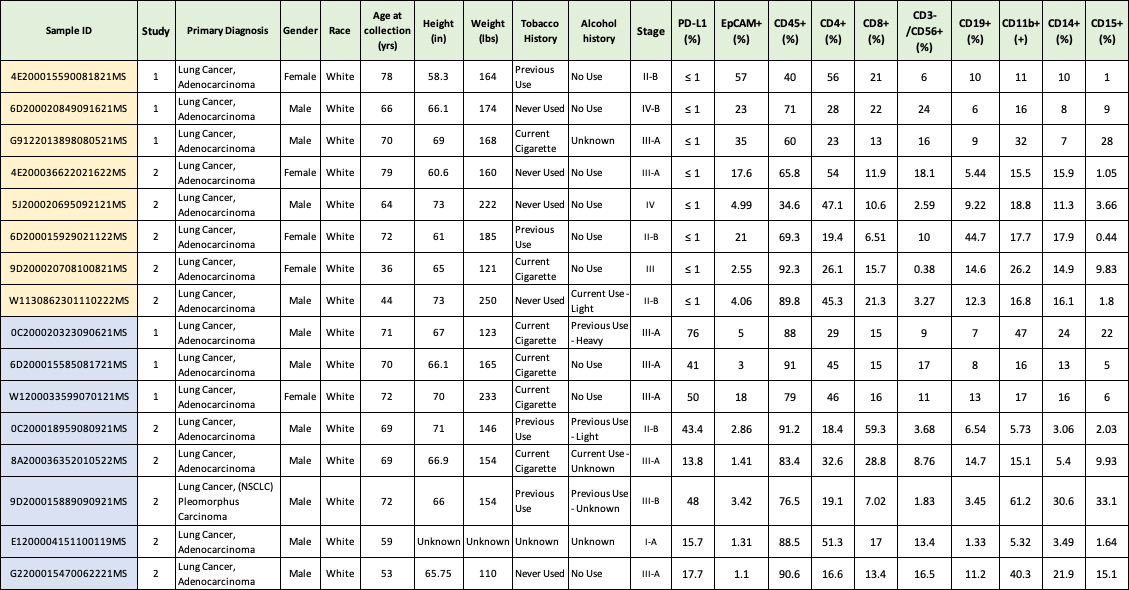


**­­­**
